# The Clinical Implementation of NEWS, SOFA, and CALL Scores in Predicting the In-Hospital Outcome of Severe or Critical COVID-19 Patients

**DOI:** 10.5152/eurasianjmed.2021.21149

**Published:** 2022-10-01

**Authors:** Zubia Jamil, Saba Samreen, Bisma Mukhtar, Madiha Khaliq, Shahid Mumtaz Abbasi, Jamal Ahmed, Tassawar Hussain

**Affiliations:** 1 Department of Medicine, Foundation University Medical College, Foundation University, DHA Phase 1 Islamabad, Pakistan; 2 Foundation University Medical College, Foundation University, DHA Phase 1 Islamabad, Pakistan; 3 Fauji Foundation Hospital, Rawalpindi, Pakistan; 4 Head of Department of Pulmonology, Fauji Foundation Hospital, Rawalpindi, Pakistan; 5 Head of Department of Medicine, Foundation University Medical College, Foundation University, DHA Phase 1 Islamabad, Pakistan

**Keywords:** COVID-19, early warning score, ROC curve, area under curve, SARS-CoV-2, organ dysfunction scores

## Abstract

**Objective:**

To date, there is no specific validated coronavirus disease 2019 score to assess the disease severity. This study aimed to evaluate the performance of the National Early Warning Score, Sequential Organ Failure Assessment, and Comorbidity-Age-Lymphocyte count-Lactate dehydrogenase scores in predicting the in-hospital outcome of critical or severe coronavirus disease 2019 patients.

**Materials and Methods:**

Single-centered analytical study was carried out in the coronavirus disease 2019 high dependency unit from April to August 2020. National Early Warning Score, Sequential Organ Failure Assessment, and Comorbidity-Age-Lymphocyte count-Lactate dehydrogenase scores were calculated for each critical to severely ill coronavirus disease 2019 patient. The diagnostic accuracy of these 3 scores in determining the in-hospital outcome of coronavirus disease 2019 patients was assessed by area under the receiver operating characteristic curve. The cut-off value of each score along with sensitivity, specificity, and positive and negative likelihood ratio were calculated by Youden index. Predictors of outcome in coronavirus disease 2019 patients were analyzed by Cox-regression analysis.

**Results:**

The area under the curve was highest for the Comorbidity-Age-Lymphocyte count-Lactate dehydrogenase score (area under the curve = 0.85) while the Sequential Organ Failure Assessment score had an area under the curve of 0.72. The cut-off values for National Early Warning Score score was 8 (sensitivity = 72.34%, specificity = 76.10%), Sequential Organ Failure Assessment score was 3 (sensitivity = 68.97%, specificity = 67.42%), and Comorbidity-Age-Lymphocyte count-Lactate dehydrogenase score was 8 (sensitivity = 88.89%, specificity = 66.67%). The pairwise comparison showed that the difference between the area under the curve of these 3 scores was statistically insignificant (*P* > .05). The rate of mortality and invasive ventilation was significantly high in groups with high National Early Warning Score, Sequential Organ Failure Assessment, and Comorbidity-Age-Lymphocyte count-Lactate dehydrogenase scores (*P* < .0001). These 3 scores, age, low platelets, and high troponin-T levels were found to be statistically significant predictors of outcome

**Conclusion:**

Comorbidity-Age-Lymphocyte count-Lactate dehydrogenase score had a good area under the curve, the highest sensitivity of its cut-off value, required only 4 parameters, and is easy to calculate so it may be a better tool among the 3 scores in outcome prediction for coronavirus disease 2019 patients.

**Main Points**

- All 3 scores (National Early Warning Score (NEWS), Sequential Organ Failure Assessment (SOFA), and Comorbidity-Age-Lymphocyte count-Lactate dehydrogenase (CALL) scores) are good prognosticators to determine the outcome of patients with coronavirus disease 2019 (COVID-19) infection
- Comorbidity-Age-Lymphocyte count-Lactate dehydrogenase score had a good area under the curve, the highest sensitivity of its cut-off value, required only 4 parameters, and is easy to calculate so it may be a better tool among the 3 scores in outcome prediction for COVID-19 patients.
- Age, platelet count, troponin T level, NEWS, SOFA, and CALL scores are found to be predictors of outcome in severe or critical hospitalized COVID-19 patients.

## Introduction

Coronavirus disease 2019 (COVID-19) infection has posed this century’s greatest challenge to humanity. With its emergence as an unexplained viral illness in Wuhan in December 2019, it spread enormously to the rest of the world.^1^ So far, COVID-19 has resulted in thousands of deaths globally. Although COVID-19 infection exhibits itself as mild upper respiratory symptoms like flu and cold-like symptoms in many patients, in others, it can present as severe respiratory tract illness or even have a fatal outcome.^2,3^ A mortality rate of 11%-62% among severely affected or critical patients with COVID-19 has been reported.^4^

With an increase in the number of COVID-19 victims and a lack of health care resources, it has become difficult to effectively manage these patients.^5^ Henceforth, triaging high-risk patients at the earliest instance and ensuring their timely access to medical intervention are of paramount importance in reducing morbidity and mortality. There are a number of parameters that were utilized initially to assess disease severity such as white blood cell count, d-dimers, and interleukin 6 levels.^6^ But none of these alone would serve as a definitive marker of disease severity and poor outcome. Similarly, there is no specific validated COVID-19 severity score in place to date. To overcome this difficulty, various centers have utilized a number of pre-existing early warning scores (EWS) used to triage patients in the emergency department.^7^ Notable among these EWS are National Early Warning Score (NEWS) and Modified Early Warning Score (MEWS).^8,9^ Similarly, Sequential Organ Failure Assessment (SOFA) score and Comorbidity-Age-Lymphocyte count-Lactate dehydrogenase (CALL) score are also utilized.^10,11^

At present numerous studies exist that assess these EWS in emergency settings. With regards to COVID-19 patients, not many studies exist that have effectively used these EWS as a predictor of patient outcome. In keeping with the lack of a validated COVID-19-specific severity assessment tool, we need to probe into the existing ones to make use of a good one among them. Therefore, the rationale of our study was to evaluate the performance of these indicators (NEWS, SOFA, and CALL scores) calculated at the time of admission in predicting in-hospital outcome of patients with critical or severe COVID-19 infection.

## Materials and Methods

### Study Design and Settings

Single-centered analytical cross-sectional study was carried out in the COVID-19 high dependency unit (COVID-19 HDU) of Fauji Foundation Hospital, Rawalpindi from Mid-April 2020 to the last week of August 2020. Fauji Foundation Hospital Rawalpindi is a large 850-bedded tertiary care hospital that was established to serve the families of retired armed people. This hospital established HDU in response to the COVID-19 pandemic which was fully equipped with all the necessary advanced facilities (invasive mechanical ventilation, continuous and bi-level airway positive pressure, 24-hour continuous oxygen delivery to patients through mask, nasal prongs, or rebreather masks), all investigational therapies (therapeutic plasmapheresis and convalescent plasma therapy), and availability of investigational pharmacological agents (remdesivir, tocilizumab, etc.) for management of severe to critical COVID-19 patients admitting in HDU.

The main objective was to evaluate the efficacy of 3 scoring systems (NEWS, SOFA, and CALL scores) calculated at the time of admission in determining the outcome of patients with severe or critical COVID-19 infection.

### Characteristics of Study Participants

This study included the following patients:

1. Patients aged >13 years with reverse transcription-polymerase chain reaction confirmed COVID-19 infection and were admitted to COVID-19 HDU due to critical or severe disease.
2. Patients with infiltrates >50% on chest x-ray ± High Resolution CT chest (HRCT) chest suggestive of extensive peripheral ground glass opacities ± blood pressure (BP) <90 mmHg with Heart rate (HR) >100/min ± respiratory rate (RR) >30/min ± SpO_2_ <90% (severe disease category).
3. Patients with evidence of acute respiratory distress syndrome or cytokine release syndrome or septic shock ± multi-organ involvement (critical disease category).

The patients aged <13 years and patients who died within 24 hours (provided their laboratory tests were not done) were excluded from the study (n = 11).

### NEWS, SOFA, and CALL Scores

1. National Early Warning Score was calculated by 7 parameters (respiratory rate, oxygen saturation, supplemental oxygen, systolic BP, pulse, temperature, and level of consciousness).^12^
2. Sequential Organ Failure Assessment score was calculated by 7 parameters (PaO_2_/FiO_2_ level, platelet count, bilirubin, creatinine, Glasgow Coma Score, mean BP + administration of vasoactive agents and mode of oxygen delivery, Continuous positive airway pressure (CPAP) or invasive ventilation.^13^
3. Comorbidity-Age-Lymphocyte count-Lactate dehydrogenase score was calculated by 4 parameters (age, co-morbidities, lactate dehydrogenase, and platelet count) (Supplementary Material NEWS score).^14^

### Methodology

The hospital developed a policy at the time of establishing COVID-19 HDU that when admitted to COVID-19 HDU, informed written consent would be signed by every patient or their relative that clinical, laboratory, and biochemical data of patients can be utilized for COVID-19-related research purposes with the following aims:

- to provide benefit to patients all over the world suffering from this disastrous disease,
- to share the experience of doctors of this hospital with other institutes of this country as well as other countries,
- to outline the effective treatment strategy of patients for management of COVID-19 infection by retrospective analyzing the collected data, and
- to update this treatment strategy time by time.

The privacy of each patient was maintained throughout the data collection and analysis. Individual identity was concealed. All the data of patients were maintained in MEDIX medical software system. Every laboratory test result can be retrieved by a specific admission record number allocated to each patient at the time of admission. Approval of this study was taken from Fauji Foundation Hospital Ethical Committee on March 15, 2020, with reference number (FF/RWP/189-03-20). National Early Warning Score, SOFA, and CALL were calculated for each critical to severely ill COVID-19 patient getting admitted to HDU. The clinical outcome (survived and non-survived and invasive and non-invasive ventilation at the time of outcome) was noted for the study cohort.

### Statistical Analysis

Statistical analysis was done with the help of MedCalc Statistical Software 19.6.4 (MedCalc Software, Ostend, Belgium). The percentages were used for qualitative variables. Ranges, means, and standard deviation were used for quantitative variables IBM Statistical Package for the Social Sciences version 26.0 software (IBM Corp., Armonk, NY, USA). The diagnostic accuracy of 3 scores (NEWS, SOFA, and CALL scores) in determining the outcome of COVID-19 patients was assessed by the area under the curve (AUC) calculated by the receiver operating characteristic (ROC) curve. The cut-off value of each score along with sensitivity, specificity, and positive and negative likelihood ratio was calculated by the Youden index (MedCalC software). This cut-off value was used to group the patients. Quantitative variables were compared by *t*-tests and qualitative variables were compared by chi-square tests. In the end, predictors of outcome in COVID-19 patients were further analyzed by Cox regression analysis.

## Results

A total of 214 patients were admitted to the HDU and 203 patients (n = 203) were included after excluding 11 patients.

The mean age was 57.14 + 15.19 (14-88) years. Among 203 patients, 77.3% (n = 157) were females and 22.7% (n = 46) were males.

The primary aim of this study was to evaluate the efficacy of the different scoring systems in determining the outcome of patients with COVID-19 infection. Three scoring systems were evaluated, namely, NEWS, SOFA, and CALL score.

In this study, we found that the presence of a co-morbid condition is the major cause of disease severity, HDU admission, and oxygen requirement among patients with COVID-19 infection. Out of 203 patients getting admission to HDU, 135 patients (66.5%) had co-morbid conditions. Only 68 patients (33.5%) did not have any co-morbid condition. The most common co-morbid condition that was present among COVID-19 patients requiring HDU admission was diabetes mellitus 52.2% (n = 106). In addition, other co-morbid conditions such as hypertension 44.8% (n = 91), chronic kidney disease 12.3% (n = 25), ischemic heart disease 8.3% (n = 17), chronic obstructive pulmonary disease 6.8% (n = 14), cerebrovascular accidents 5.9% (n = 12), congestive cardiac failure 4.9% (n = 10), chronic liver disease 3.9% (n = 8), and the presence of malignancy and use of immune-suppressive agents 2.9% (n = 6) were also present among these patients.

The vital signs, laboratory parameters, and modes of supplemental oxygen required by 203 patients in the study cohort of COVID-19 infection needed for the calculation of 3 scores (NEWS, SOFA, and CALL) are shown in Table 1.

### ROC Curve for NEWS, SOFA, and CALL Score

**Figure 1.**
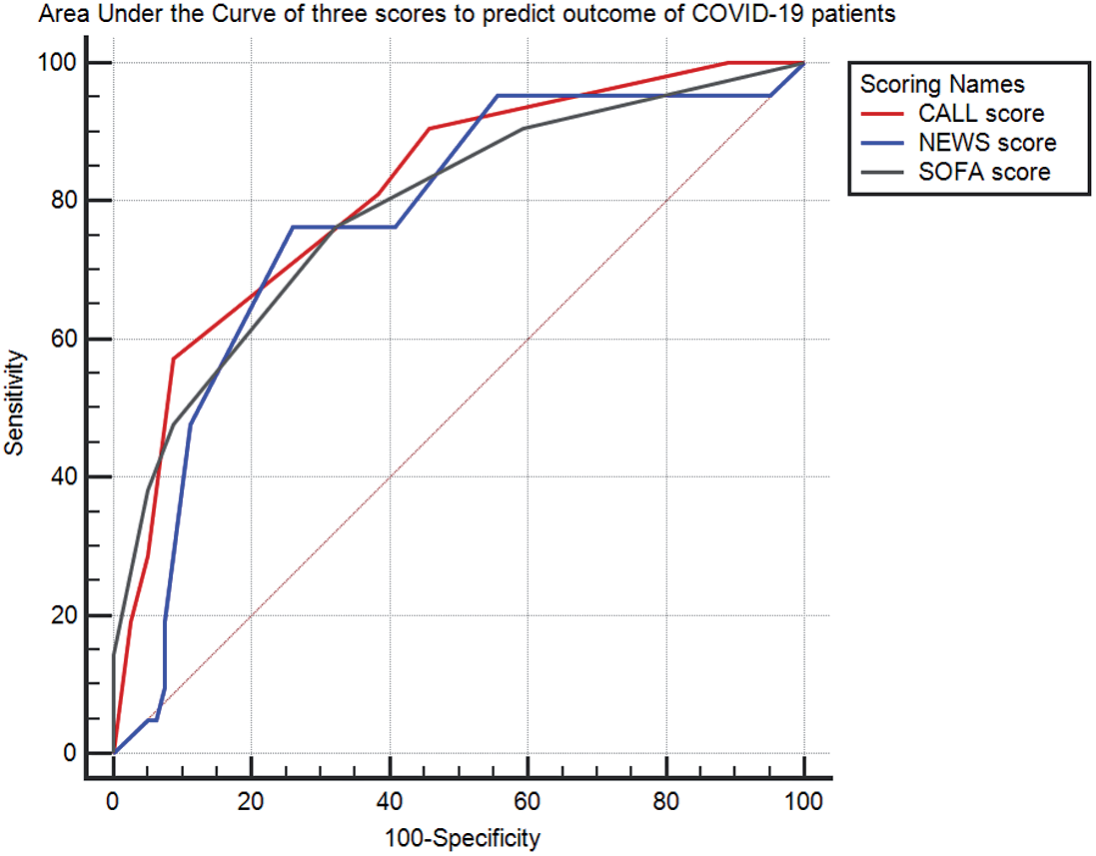
The AUC calculated by ROC showing 3 scores (CALL, NEWS, and SOFA) to predict the outcome in patients with severe to critical COVID-19 patients. AUC, area under the curve; ROC, receiver operating curve; NEWS, National Early Warning Score; SOFA, Sequential Organ Failure Assessment; CALL, Comorbidity-Age-Lymphocyte count-Lactate dehydrogenase; COVID-19; coronavirus disease 2019.

The AUC calculated by ROC was used to determine the efficacy of each score as an outcome predictor in patients with COVID-19 infection. Youden index was used to calculate the cut-off value for each score with sensitivity, specificity, positive likelihood ratio, and negative likelihood ratio. The AUC calculated by ROC of 3 scores (CALL, NEWS, and SOFA) to predict the outcome in patients with severe or critical COVID-19 patients is shown in Figure 1.

The mean NEWS score was 7.18 + 2.77 (2-14) at the time of admission. Sequential Organ Failure Assessment score was 3.41 + 1.53 (2-10) while CALL score was 8.61 + 2.19 (5-13). The AUC was highest for the CALL score (AUC = 0.85) while the SOFA score had an AUC of only 0.72. The AUC for each score with 95% CI and the cut-off value calculated by Youden index with sensitivity, specificity, positive likelihood ratio, and negative likelihood ratio for that cut-off value in relation to *P*-value are shown in Table 2.

The pairwise comparison of AUC of scores was also done. It showed that the difference between AUC of CALL and NEWS score was 0.04 (standard error (SE) = 0.07, 95% CI: −0.09 to 0.18, *P* = .54), the difference between AUC of CALL and SOFA score was 0.02 (SE = 0.07, 95% CI: −0.16 to 0.15, *P* = .69), and the difference between AUC of NEWS and SOFA score was 0.02 (SE = 0.07, 95% CI: −0.12 to 0.16, *P* = .82).

### Clinical Outcomes According to NEWS, SOFA, and CALL Scores

Among 203 patients admitted to HDU, 75.9% (n = 154) patients survived and 24.1% (n = 49) patients died due to COVID-19 infection. The respiratory failure 7.8% (n = 16) was the most common cause of death in these patients followed by pulmonary embolism 6.8% (n = 14), septic shock 6.4% (n = 13), and arrhythmias 2.9% (n = 6).

The study cohort was divided into 2 groups according to the cut-off value calculated by the Youden index. The cut-off value for NEWS and CALL score was 8 and for the SOFA score was 3. The analysis of survival percentages and modes of ventilation at the time of outcome in groups of study cohort according to the cut-off values of NEWS, SOFA, and CALL scores is shown in Table 3.

Regression analysis was used to determine the single determinants influencing the outcome of patients with COVID-19 infection. First, all variables were tested with univariate analysis then only those variables found to be predictors of outcome were further tested by multivariate analysis. Omnibus tests showed that the model was statistically fit for analysis (chi-square test = 29.87, *P* = .01) as the model covered 22%-34% variation of variables (Cox and Snell pseudo’s R^2^ and Nagelkerke pseudo’s *R* ^2^, respectively) and classified 75.4% of cases. Among quantitative variables, age, platelets, troponin T, NEWS, SOFA, and CALL scores were found to be statistically significant predictors of outcome while among qualitative variables, the presence of comorbid conditions and groups of NEWS, SOFA, and CALL scores according to cut-off value were found to be independent predictors of outcome of COVID-19 patients. The various predictors that had a statistically significant effect on the outcome of patients in the study cohort are shown in Table 4.

## Discussion

It has been just a few months back that the World Health Organization declared COVID-19 as a pandemic on March 11, 2020.^1^ Ever since then, it has tremendously burdened the health infrastructure with unmet medical demands and also crippling economics. The spectrum of COVID-19 ranges from mild symptoms to critical forms leading to increased morbidity and mortality. Early and appropriate selection of high-risk patients with poor outcomes is of paramount importance. This study focuses on determining the efficacy of the NEWS, SOFA, and CALL scores in determining the outcome of COVID-19.

### NEWS Score

It was first developed in England in 2012 to replace the locally existing EWS and has now become a globally accepted tool.^15^ For COVID-19 patients, the advantage of the NEWS score is the inclusion of more appropriate parameters including SpO_2_ and respiratory indices.^12^ It is a good predictor of mortality and deterioration both in prehospital and in-hospital setups.^16^ In COVID-19 contexts, little is known about its usefulness in predicting clinical outcomes. In 1 study conducted by Wellbelove et al,^17^ NEWS score had a poor prediction of 30-day mortality in patients with severe COVID-19 (AUC = 0.48, 95% CI; 0.23-0.73, *P *= .89). In another large multi-centered study done on nearly 800 patients conducted by Mitacchione et al,^18^ the authors used NEWS score to determine the disease severity in COVID-19 patients to find the effects of statins therapy on the outcomes of these patients. In our study, the AUC of NEWS score is 0.78 for predicting the clinical outcomes in COVID-19 patients depicting moderate utility of this score for moderate-severe COVID-19. The cut-off value of the NEWS score was obtained by the Youden index and it was 8. At a cut-off value of 8, it has a sensitivity of 72.34 and specificity of 76.10. The percentage of patients who did not survive with NEWS score >8 was 17.7% versus only 6.9% non-survived percentages of patients with a NEWS score <8 (*P* < .05). With respect to the result as an invasive or non-invasive ventilation mode, among patients with a NEWS score >8, 7.9% required invasive ventilation and only 4.4% required invasive ventilation among patients with a score below 8 (*P* < .05).

### SOFA Score

It was first developed in 1994 and encompasses diverse parameters of multiple organ systems. It is a widely validated tool for critical diseases,^19^ but it is not acquired as quickly as the other 2 EWS because acquiring lab parameters takes time. The increasing score has a good correlation with increasing mortality.^20^ In the context of COVID-19, the SOFA score also had an acceptable utility (AUC = 0.6) for outcome in a previous study.^21^ In our study, similar results were obtained. The AUC of SOFA for prediction of the non-survived outcome is 0.72 and with a cut-off of 3, its sensitivity of 68.97%, and specificity of 67.42%. Although this sensitivity and specificity are slightly lower than the NEWS score, the AUC for the prediction of outcome is nearly the same for the NEWS and SOFA score (AUC = 0.78 vs. 0.72, *P* > .05). The mortality rate in patients with SOFA score >3 was 19.7%, while only 4.4% of patients died with a score <3 (*P* < .05). Similarly, only a trivial portion of patients, that is, 0.9% required invasive ventilation with a score <3 compared to 7.4% who required invasive ventilation with a score >3 (*P* < .05).

### CALL Score

The CALL score has been devised as a means to anticipate disease progression in COVID-19. Though it encompasses 4 simple parameters, it is unique as it does not take the respiratory parameters despite the fact that up to 15% of patients have florid respiratory involvement ranging from interstitial pneumonia to respiratory failure.^22^ In our study, the AUC of the CALL score is 0.85. Similarly, another study done by Ji et al^11^ found the CALL score as a novel scoring model to predict the outcome and outline the management plan for COVID-19 patients. They found an AUC of above 0.90 and at a cut-off value of 6, positive predictive value was around 50%, and a very high negative predictive value of nearly 99%. In our study, the cut-off value was calculated for the CALL score and it was 8. At this cut-off value, it has the highest sensitivity of 88.89% compared to the sensitivity of cut-off values for the NEWS and SOFA scores. Likelihood ratios for this cut value (8) were also significant with a positive likelihood ratio >1 (2.67) and a negative-positive likelihood ratio <1 (0.17) (*P* < .0001). This is evident from Table 3 which showed that the greater portion of patients required invasive ventilation and they died whose CALL score was >8.

In our study, AUC estimation showed that all 3 scoring systems performed well as outcome predictors in patients with COVID-19 but the CALL score outperformed the other 2 (AUC = 0.85). It is worth mentioning here that pairwise comparisons among these scores (CALL ~ NEWS, CALL ~ SOFA, and NEWS ~ SOFA) did not reveal that any of these 3 scoring systems are better than the others (*P *> .05), but as CALL score had AUC >0.80, its cut-off value has good sensitivity, it required only 4 parameters, and it is very easy to calculate, so CALL score may be a better tool among the 3 scoring systems in outcome prediction for moderate to severe COVID-19.

It has been observed that the presence of co-morbid conditions has been associated with the unfavorable course for COVID-19 patients. In our study, 66.5% of patients had co-morbid conditions leading to increased severity of disease and more HDU admission. The commonest co-morbidity in our cohort was diabetes (52.2%) followed by hypertension, chronic kidney disease, and ischemic heart disease (44.8%, 12.3%, and 8.3%, respectively). The study by Tian et al^23^ shows that 45% of severe COVID-19 patients had co-morbidities. Other studies also show a similar proportion of comorbidities in patients with COVID-19.^24,25^

In our study, patients of older age were at higher odds of a poor outcome (OR 1.15, *P* = .01). Furthermore, low platelets, high Trop-T, and the presence of co-morbidity are found as significant predictors of outcomes. Same observations have been made in other studies as well.^24-27^ Similarly, the presence of comorbidities and high NEWS, SOFA, or CALL scores at the time of assessment also had a significant impact on the patient’s outcome (odds ratio = 1.21, 1.80, and 2.24, respectively).

Although it is a single-centered study and hence would greatly limit the generalizability of the results to other parts of the world, this study is among the first to determine the predictive efficacy and compare various COVID-19 severity scoring systems in moderate to severely affected patients.

In conclusion, all 3 scores (NEWS, SOFA, and CALL scores) are all good prognosticators to determine the outcome of patients with COVID-19 infection, but since the CALL score requires only 4 parameters and it is very easy to calculate, CALL score may be a better tool among the 3 scoring systems in outcome prediction for moderate to severe COVID-19. Until a specific COVID-19 score becomes available, these scores may be used to help identify high-risk patients and for the timely allocation of available medical resources.

## Figures and Tables

**Table 1.** Table Showing the Vital Signs, Laboratory Test Results, and Frequency of Modes of Supplemental Oxygen Delivered to 203 Patients in the Study Group.

**Variables**	**Mean + SD**
Age (years)	55.43 + 15.36
BP systolic (mmHg)	136.88 + 25.87
BP diastolic (mmHg)	77.24 + 14.22
Pulse	97.88 + 17.22
Respiratory rate	28.76 + 5.36
Temperature	99.46 + 0.67°F
Oxygen saturation	86.22% + 14.12%
Hemoglobin (g/dL)	11.38 + 2.24
WCC × 10^3^ cells/L	12.64 + 5.27
Lymphocytes (%)	1.59 + 0.76
Platelets × 10^3^ cells/L	255.33 + 98.23
Bilirubin (umol/L)	11.70 + 4.36
Creatinine	262.28 + 61.83
PT	1.81 + 0.35
aPTT	2.01 + 0.48
LDH (U/L)	492.13 + 155.56
PaO_2_/FiO_2_ (mmHg)	361.44 + 81.44
**Supplemental oxygen**	
Oxygen <10 L/min (nasal prongs or mask)	51.7% (n = 105)
Oxygen >10 L/min (nasal prongs or mask)	7.4% (n = 15)
Rebreather mask	10.8% (n = 22)
BiPAP	13.8% (n = 28)
Invasive mechanical ventilation	12.3% (n = 25)

SD, standard deviation; BP, blood pressure; WCC, white cell count; PT, prothrombin time; aPTT, activated partial thromboplastin time; LDH, lactate dehydrogenase; BiPAP, bilevel positive airawy pressure.

The mean with standard deviation is used for expression of quantitative variables and percentages are used for expression of qualitative variables

**Table 2.** Table Showing the Area Under the Curve, Cut-Off Value of Each Score with Sensitivity, Specificity, Positive Likelihood Ratio, and Negative Likelihood Ratio

**Scores**	**AUC**	**95% CI**	**Cut-Off Value**	**Sensitivity**	**Specificity**	**+LR**	−**LR**	***P***
NEWS	0.78	0.72-0.83	>8	72.34	76.10	3.01	0.36	<.0001
SOFA	0.72	0.63-0.80	>3	68.97	67.42	2.12	0.46	<.0001
CALL	0.85	0.79-0.90	>8	88.89	66.67	2.67	0.17	<.0001

AUC, area under the curve; +LR, positive likelihood ratio; −LR, negative likelihood ratio; NEWS, National Early Warning Score; SOFA, Sequential Organ Failure Assessment; CALL, Comorbidity-Age-Lymphocyte count-Lactate dehydrogenase.

**Table 3.** Table Showing the Analysis of Survival Percentages and Modes of Ventilation at the Time of Outcome in Groups of Study Cohort According to Cut-Off Value of NEWS, SOFA, and CALL scores

**Scores**	**Groups**	**Survived**	**Non-survived**	**Chi-Square**	***P***
NEWS	<8	58.1% (118)	6.9% (14)	36.50	.000
	>8	17.7% (36)	17.2% (35)		
SOFA	<3	66% (134)	4.4% (9)	11.92	.001
	>3	9.9% (20)	19.7% (40)		
CALL	<8	47.2% (96)	1.9% (4)	36.01	.000
	>8	23.6% (48)	15.8% (32)		
		**Non-invasive Ventilation**	**Invasive ventilation**		
NEWS	<8	56.2% (114)	4.4% (9)	28.94	.000
	>8	24.6% (50)	7.9% (16)		
SOFA	<3	33.5% (68)	0.9% (2)	64.40	.000
	>3	16.3% (33)	7.4% (15)		
CALL	<8	44.8% (91)	3.9% (8)	16.57	.002
	>8	30.1% (61)	6.9% (14)		

NEWS, National Early Warning Score; SOFA, Sequential Organ Failure Assessment; CALL, Comorbidity-Age-Lymphocyte count-Lactate dehydrogenase.

**Table 4.** Cox Regression Analysis Showing Variables in Predicting Survival of Patients with COVID-19 Infection

**Variables**	**OR (95% CI)**	***P***
Age (years)	1.15 (1.04-1.20)	.01
Platelets × 10^3^ cells/L	0.99 (0.94-0.98)	.01
Trop T	0.21 (0.04-4.42)	.01
Presence of co-morbidities	0.23 (0.06-0.77)	.00
NEWS score	1.21 (1.01-1.45)	.03
SOFA score	1.80 (1.29-2.51)	.00
CALL score	2.24 (1.68-2.99)	.00
Groups of NEWS score (>8)	0.20 (0.08-0.53)	.00
Groups of SOFA score (>3)	0.20 (0.08-0.54)	.00
Groups of CALL score (>8)	0.06 (0.02-0.18)	.00

Trop T, troponin T; OR, odds ratio; NEWS, National Early Warning Score; SOFA, Sequential Organ Failure Assessment; CALL, Comorbidity-Age-Lymphocyte count-Lactate dehydrogenase.

**Table:** 

**NEWS score Table**							
**Physiological Parameters**	**3**	**2**	**1**	**0**	**1**	**2**	**3**
Respiratory rate	<8		9-11	12-20		21-24	>25
Oxygen saturations (%)	<91	92-93	94-95	>96			
Supplemental oxygen		yes		no			
Systolic BP (mmHg)	<90	90-100	101-110	111-219			>220
Pulse (bpm)	<40		41-50	51-90	91-110	111-130	>131
Temperature (^O^C)	<35		35.1-36.0	36.1-38.0	38.1-39.0	>39.1	
Level of consciousness				A			V,P or U

**Table:** 

**NEWS scores**	**Clinical Risk**
1-4	Low
5-6 (RED score)	Medium
>7	High

**Table:** 

**SOFA score Table**					
	**0**	**1**	**2**	**3**	**4**
PaO_2_/FiO_2_ level; mmHg	>400	300-399	200-199	100-199 and Mechanically ventilated	<100 and Mechanically ventilated
Bilirubin; umol/L	<20	20-32	33-101	102-204	>205
Mean arterial pressure or administration of vasoactive agents	No hypotension	MAP <70	Dopamine <5, or Dobutamine (any dose)	Dopamine >5, or Epinephrine < 0.1, or Norepinephrine < 0.1	Dopamine >15, or Epinephrine > 0.1, or Norepinephrine > 0.1
Platelets x 10^3^/uL	>150	100-149	50-99	20-49	<20
GCS score	15	13-14	10-12	6-9	<6
Creatinine; umol/L)	<110	110-170	171-299	300-440	>440

**Table:** 

**SOFA scores**	**Mortality**
0-1	0.0%
2-3	6.4%
4-5	20.2%
6-7	21.5%
8-9	33.3%
10-11	50.0%
12-14	95.2%
>14	95.2%

**Table:** 

**CALL score Table**				
**Parameters**	**+1**	**+2**	**+3**	**+4**
Co-morbidities	None			>1
Age	< 60		>60	
Lymphocytes	>1000		<1000	
LDH	< 250	250-500	>500	

**Table:** 

**CALL scores**	**Clinical Risk**
<6	Low
>6	High

## Supplementary Material

National Early Warning Score (NEWS score) was calculated by 7 parameters (Respiratory rate, oxygen saturations, supplemental oxygen, systolic BP, pulse, temperature and level of consciousness)

**INTERPRETATION:**

**Sequential Organ Failure Assessment** (SOFA score) was calculated by 7 parameters (PaO_2_/FiO_2_ level, platelet count, bilirubin, creatinine, Glasgow Coma Score, mean blood pressure + administration of vasoactive agents and mode of oxygen delivery; CPAP or invasive ventilation)

**INTERPRETATION:**

**CALL** score 4 parameters (age, co-morbidities, LDH and platelet count)

**INTERPRETATION:**

## References

[1] Phelan AL Katz R Gostin LO . The novel coronavirus originating in Wuhan, China: challenges for global health governance. JAMA. 2020;323(8):709 710. 10.1001/jama.2020.1097) 31999307

[2] Chen N Zhou M Dong X et al. Epidemiological and clinical characteristics of 99 cases of 2019 novel coronavirus pneumonia in Wuhan, China: a descriptive study. Lancet. 2020;395(10223):507 513. 10.1016/S0140-6736(20)30211-7) 32007143 PMC7135076

[3] Yang X Yu Y Xu J et al. Clinical course and outcomes of critically ill patients with SARS-CoV-2 pneumonia in Wuhan, China: a singlecentered, retrospective, observational study. Lancet Respir Med. 2020;8(5):475 481. 10.1016/S2213-2600(20)30079-5) 32105632 PMC7102538

[4] Weiss P Murdoch DR . Clinical course and ­mortality risk of severe COVID-19. Lancet. 2020;395(10229):1014 1015. 10.1016/S0140-6736(20)30633-4) 32197108 PMC7138151

[5] Ji Y Ma Z Peppelenbosch MP Pan Q . Potential association between COVID-19 mortality and health-care resource availability. Lancet Glob Health. 2020;8(4):e480. 10.1016/S2214-109X(20)30068-1) PMC7128131 32109372

[6] Rothan HA Byrareddy SN . The epidemiology and pathogenesis of coronavirus disease (COVID-19) outbreak. J Autoimmun. 2020;109:102433. 10.1016/j.jaut.2020.102433) 32113704 PMC7127067

[7] Hu H Yao N Qiu Y . Predictive value of 5 early warning scores for critical COVID-19 patients. Disaster Med Public Health Prep. 2022;16(1):232 239. 10.1017/dmp.2020.324) 32900406 PMC7596567

[8] Smith GB Prytherch DR Meredith P Schmidt PE Featherstone PI . The ability of the National Early Warning Score (NEWS) to discriminate patients at risk of early cardiac arrest, unanticipated intensive care unit admission, and death. Resuscitation. 2013;84(4):465 470. 10.1016/j.resuscitation.2012.12.016) 23295778

[9] Hammond NE Spooner AJ Barnett AG Corley A Brown P Fraser JF . The effect of implementing a modified early warning scoring (MEWS) system on the adequacy of vital sign documentation. Aust Crit Care. 2013;26(1):18 22. 10.1016/j.aucc.2012.05.001) 22652368

[10] Khwannimit B Bhurayanontachai R Vattanavanit V . Comparison of the accuracy of three early warning scores with SOFA score for predicting mortality in adult sepsis and septic shock patients admitted to intensive care unit. Heart Lung. 2019;48(3):240 244. 10.1016/j.hrtlng.2019.02.005) 30902348

[11] Ji D Zhang D Xu J et al. Prediction for progression risk in patients with COVID-19 pneumonia: the CALL score. Clin Infect Dis. 2020;71(6):1393 1399. 10.1093/cid/ciaa414) 32271369 PMC7184473

[12] The Calculator. National Early Warning Score [NEWS] calculator. Available at: https://www.thecalculator.co/health/National-Early-Warning-Score-(NEWS)-Calculator-843.html Accessed 13th January,2021.

[13] MD+calc. Sequential Organ Failure Assessment (SOFA) score. Available at: https://www.mdcalc.com/sequential-organ-failure-assessment-sofa-score Accessed 20th January,2021.

[14] CALL score. Available at: https://www.rccc.eu/COVID/CALL.html Accessed 22nd January, 2021.

[15] Jones M NEWSDIG: The national early warning score development and implementation group. Clinmed. 2012;12(6):501. 10.7861/clinmedicine.12-6-501 PMC5922584 23342400

[16] Silcock DJ Corfield AR Gowens PA Rooney KD . Validation of the National Early Warning Score in the prehospital setting. Resuscitation. 2015;89:31 35. 10.1016/j.resuscitation.2014.12.029) 25583148

[17] Wellbelove Z Walsh C Perinpanathan T Lillie P Barlow G . Comparing the 4C mortality score for COVID-19 to established scores (CURB65, CRB65, qSOFA, NEWS) for respiratory infection patients. J Infect. 2021;82(3):414 451. 10.1016/j.jinf.2020.10.015) PMC7585728 33115655

[18] Mitacchione G Schiavone M Curnis A et al. Impact of prior statin use on clinical outcomes in COVID-19 patients: data from tertiary referral hospitals during COVID-19 pandemic in Italy. J Clin Lipidol. 2021;15(1):68 78. 10.1016/j.jacl.2020.12.008) 33390341 PMC7833194

[19] Vincent JL de Mendonça A Cantraine F et al. Use of the SOFA score to assess the incidence of organ dysfunction/failure in intensive care units: results of a multicenter, prospective study. Working group on “sepsis-related problems” of the European Society of Intensive Care Medicine. Crit Care Med. 1998;26(11):1793 1800. 10.1097/00003246-199811000-00016) 9824069

[20] Moreno R Vincent JL Matos R et al. The use of maximum SOFA score to quantify organ dysfunction/failure in intensive care. Results of a prospective, multicentre study. Working Group on Sepsis related Problems of the ESICM. Intensive Care Med. 1999;25(7):686 696. 10.1007/s001340050931) 10470572

[21] Raschke RA Agarwal S Rangan P Heise CW Curry SC . Discriminant Accuracy of the SOFA Score for Determining the Probable Mortality of Patients With COVID-19 Pneumonia Requiring Mechanical Ventilation. JAMA. 2021;325(14):1469 1470. 33595630 10.1001/jama.2021.1545 PMC7890534

[22] Stawicki SP Jeanmonod R Miller AC et al. The 2019-2020 novel coronavirus (severe acute respiratory syndrome coronavirus 2) pandemic: a joint American college of academic international medicine-world academic council of emergency medicine multidisciplinary COVID-19 working group consensus paper. J Glob Infect Dis. 2020;12(2):47 93. 10.4103/jgid.jgid_86_20) 32773996 PMC7384689

[23] Tian W Jiang W Yao J et al. Predictors of mortality in hospitalized COVID‐19 patients: a systematic review and meta-analysis. J Med Virol. 2020;92(10):1875 1883. 10.1002/jmv.26050) 32441789 PMC7280666

[24] Wang D Hu B Hu C et al. Clinical characteristics of 138 hospitalized patients with 2019 novel coronavirus-infected pneumonia in Wuhan, China. JAMA. 2020;323(11):1061 1069. 10.1001/jama.2020.1585) 32031570 PMC7042881

[25] Guan WJ Liang WH Zhao Y et al. Comorbidity and its impact on 1590 patients with COVID-19 in China: a nationwide analysis. Eur Respir J. 2020;55(5), 2001227. 10.1183/13993003.00547-2020) 32217650 PMC7098485

[26] Lippi G Lavie CJ Sanchis-Gomar F . Cardiac ­troponin I in patients with coronavirus disease 2019 (COVID‐19): evidence from a meta-­analysis. Prog Cardiovasc Dis. 2020;63(3):390 391. 10.1016/j.pcad.2020.03.001) 32169400 PMC7127395

[27] Shi S Qin M Shen B et al. Association of cardiac injury with mortality in hospitalized patients with COVID‐19 in Wuhan, China. JAMA Cardiol. 2020;5(7):802 810. 10.1001/jamacardio.2020.0950) 32211816 PMC7097841

